# The influence of behavioural and psychological factors on medication adherence over time in rheumatoid arthritis patients: a study in the biologics era

**DOI:** 10.1093/rheumatology/kev105

**Published:** 2015-05-12

**Authors:** Catharine Morgan, John McBeth, Lis Cordingley, Kath Watson, Kimme L. Hyrich, Deborah P. M. Symmons, Ian N. Bruce

**Affiliations:** ^1^Arthritis Research UK Centre for Epidemiology, Centre for Musculoskeletal Research, Institute of Inflammation and Repair, University of Manchester, Manchester Academic Health Science Centre, Manchester,; ^2^Research Institute for Primary Care & Health Sciences, Keele University, Keele,; ^3^Institute of Inflammation & Repair, University of Manchester, Manchester Academic Health Science Centre and; ^4^NIHR Manchester Musculoskeletal Biomedical Research Unit, Central Manchester University Hospital NHS Foundation Trust, Manchester Academic Health Science Centre, Manchester, UK

**Keywords:** rheumatoid arthritis, medication adherence, biologic therapy, illness perceptions, medication beliefs, epidemiology

## Abstract

**Objectives.** To investigate levels of self-reported adherence to biologic treatment and establish the contribution of demographic, physical and psychological factors to biologic medication adherence in an RA cohort.

**Methods.** Adalimumab-treated patients were recruited through the British Society for Rheumatology Biologics Register for RA between May 2007 and April 2009. Demographic and baseline psychological measures including illness and medication beliefs were collected. Disease activity (28-item DAS), physical function (HAQ) and quality of life (36-item Short Form Health Survey) were also measured at baseline and at 6, 12 and 18 months. Adherence was assessed at each follow-up using the patient self-completed Compliance Questionnaire for Rheumatology (CQR). Multilevel mixed effects modelling analysis was performed to investigate predictors of adherence.

**Results.** Of the 329 Adalimumab-treated patients included, low adherence (CQR score <65) was reported in 23%, with 41% reporting low adherence at at least one time point. After controlling for age and disease duration, factors independently predictive of increased adherence were increased belief in medication necessity, with baseline effect diminishing over time [β coefficient 1.68 (s.e. 0.19), *P* = 0.0001], lower medication concerns [0.50 (0.15), *P* = 0.001], with this effect remaining throughout follow-up, increased professional or family member support [0.81 (0.32), *P* = 0.01], strong views of illness being chronic [0.32 (0.14), *P* = 0.025] and increased treatment control [0.41 (0.19), *P* = 0.032].

**Conclusion.** Wider recognition of the importance of psychological factors, particularly medication beliefs, in driving medication adherence could have substantial clinical and health economic benefits in RA. The psychological factors we have identified are putative targets for strategies to improve adherence in RA.

Rheumatology key messages

- A quarter of RA patients show only low to moderate adherence to adalimumab.
- Illness and treatment beliefs are the major influences on adherence to adalimumab among RA patients.
- Higher perceived support from health professionals and family may improve adherence to adalimumab in RA.

## Introduction

Medication adherence is defined as the extent to which a patient’s behaviour in taking their medication corresponds to agreed recommendations by their health care provider [1]. More than one-third of therapies are not taken as recommended, irrespective of the seriousness of disease or condition [2]. There is increasing recognition of lower adherence even in symptomatic diseases such as RA with medication adherence rates reported between 55% and 96% [3–6]. Little is known about biologic drug adherence in RA, and studies are further limited as adherence rates tend to be derived from proxy measures, including medication persistence (time from prescription initiation to prescription discontinuation), drug survival or medication possession ratios from administrative claims data [7–11]. With the wider use of biologic therapy in RA, together with reported low medication possession ratios and persistence rates suggested in RA in general, there is a clear need to investigate adherence rates of biologic therapy in real-world practice.

The impact of medication non-adherence may be considerable; adherent patients have more favourable outcomes [12], including better disease control, higher remission rates and improved physical function [13, 14], as well as lower rates of disease progression and escalation to further aggressive treatment [15, 16]. Biologic therapies also have high lifetime costs to the health care system [17], and lower persistence to biologic therapy is associated with higher non-pharmacy costs [18].

Adherence is recognized to require sustained behavioural change, influenced by both environmental and psychological factors. In RA, influences on adherence include age [10, 14], ethnicity [19], socio-economic factors [5, 20], complexity of treatment [21] and RA disease-specific factors such as inflammatory markers (ESR) and disease activity; however, it is important to note that findings are not consistent across studies. In other disease groups, the important influences of patients’ illness perception and medication beliefs on adherence behaviour [22, 23], as directed by the extended Self-Regulatory Common Sense Model (SR-CSM) of illness [24] and treatment [25] have been highlighted. According to the SR-CSM, an individual’s illness perception, such as beliefs about disease consequences or perceived personal control, influence coping, including self-management strategies, in response to the perception of a health threat [24]. A further extension to this model is the necessity-concern framework [25], suggesting patients’ beliefs about their medication, including the perceived need for and/or concerns about medication use, are an influence on medication adherence behaviour.

The aim of the current study was to investigate the level of adherence to a biologic therapy longitudinally using an RA-specific measure of adherence. We also sought to determine the relative contribution of demographic factors, RA disease-specific influences and psychological behavioural influences on adherence in a single prospective cohort.

## Methods

### Setting and recruitment

Patients were recruited through the British Society for Rheumatology Biologics Register for RA (BSRBR-RA), a UK-wide RA prospective observational cohort study established in 2001 to monitor the long-term safety of biologic therapy use in RA [26, 27]. Patient eligibility for a biologic drug followed existing National Institute for Health and Care Excellence criteria which were: satisfying 1987 ACR classification criteria for RA [28], having active disease with a 28-item DAS (DAS28) score [29] >5.1 and failing two or more previous synthetic DMARDs (sDMARDs), including MTX. All patients were clinically diagnosed by their treating physician. It was the physician’s decision to initiate a biologic, as well as the chosen biologic therapy, and no specific exclusion criteria applied. The level of provision of information about the biologic therapy or education about its use was based on the centre’s routine practice and not contingent upon the individual’s participation in the study. This substudy focused on patients starting s.c. adalimumab (ADA) as their first biologic drug between May 2007 and April 2009. This was the main biologic drug under active recruitment to the study at this time. All patients gave written informed consent prior to inclusion and this study was approved by the North West Research Ethics Committee (REC:MREC 00/8/053).

### Data collection

#### Baseline (start of treatment)

After written informed consent was obtained, the local centre provided the year of diagnosis, 1987 ACR criteria fulfilled and the DAS28 score [29]. A dichotomized variable was derived for an acute phase response from age- and gender-adjusted upper limits of the normal range of ESR [30] or CRP [31]. Patients provided date of birth, gender, ethnicity, work status, smoking status and postcode for calculation of socio-economic status using the country-specific Index of Multiple Deprivation [32–34]. Patients also returned the following self-reported questionnaires: Stanford HAQ [35]; 36-item Short Form Health Survey (SF-36) [36] and the EuroQol five-dimensions questionnaire (EQ-5D) [37] using transformed weighted health state index scores [38] and dichotomizing (≥0.516). Additional measures used were the Revised Illness Perception Questionnaire (IPQ-R) [39], capturing illness beliefs based on the SR-CSM [24], where higher scores across domains are indicative of a greater sense of symptomology, the acute long-lasting and cyclical nature of the disease, understanding and ultimate consequence of the disease, personal treatment control and a higher emotional state. The higher scores on the Beliefs about Medicines Questionnaire [25] are indicative of a stronger feeling of medication need and concern towards medication use. Also used were the Hospital Anxiety and Depression Scale (HADS) [40] and an adaptation of the Daily Coping Inventory, which assesses the level of coping based on the number of preclassified strategies adopted by an individual [41].

#### Follow-up

Patients were mailed follow-up questionnaires, including the HAQ and SF-36, at 6-monthly intervals (6, 12 and 18 months). Postal reminders were sent at 2 weeks and a further reminder with repeat questionnaire at 4 weeks for baseline or follow-up non-returners. The local centre provided the DAS28 score.

#### Adherence

As the main outcome measure, adherence data were collected at 6, 12 and 18 months after the baseline measures were recorded. The 19-item Compliance Questionnaire for Rheumatology (CQR) [42] has been validated against other adherence measures [5, 6, 20], including the Medication Events Monitoring system [43]. Patients rate their agreement with 19 statements using a 4-point Likert scale. The adjusted total score ranges from 0 to 100 (100 indicating the highest possible adherence) and is used as a continuous scale.

## 

### Analysis

After assessing for attrition and assigning missing data as missing at random, appropriate application of Multiple Imputation for Chained Equations [44] for 40 imputations provided the imputed datasets. Complete case (CC) results are presented with reference to imputed findings where appropriate.

Multilevel mixed effects modelling analysis was performed on 329 individuals with CC baseline data to describe the longitudinal relationship between adherence (CQR score) and potential predictors [45]. This allowed for within- and between-patient and follow-up variability of adherence score over time. In addition, a mixed model approach assumes data are missing at random, rather than missing completely at random. This allows all individuals to be retained and their available data utilized, whether or not complete, to address potential bias issues. Sixty-two per cent of the total variance in the CQR score was represented at patient level (unconditional model: intraclass correlation 0.62, between-patient variance 67.29, time variance 40.94).

#### Univariate analysis

Random intercept models were applied to each predictor variable to determine their prognostic value, controlling for age at follow-up, gender, social deprivation and disease duration. Interaction terms between the effects of each predictor on adherence at each follow-up period were retained where significant. Each significant predictor from the intercept models (*P* < 0.05) were further modelled by including their random slope and then tested using likelihood ratio tests. The inclusion of random slopes was not warranted for all predictors.

#### Multivariate analysis

A multivariate model was determined by adding and retaining any significant univariate factors with applicable interactions (*P* < 0.05). Models returned maximum likelihood estimators and their efficiency was assessed using the Akaike’s Information Criterion. Further diagnostics were performed by inspection of normality of residuals. All analysis was performed using STATA 11.2 (StataCorp, College Station, TX, USA).

## Results

### Response rates

**Fig. 1:**
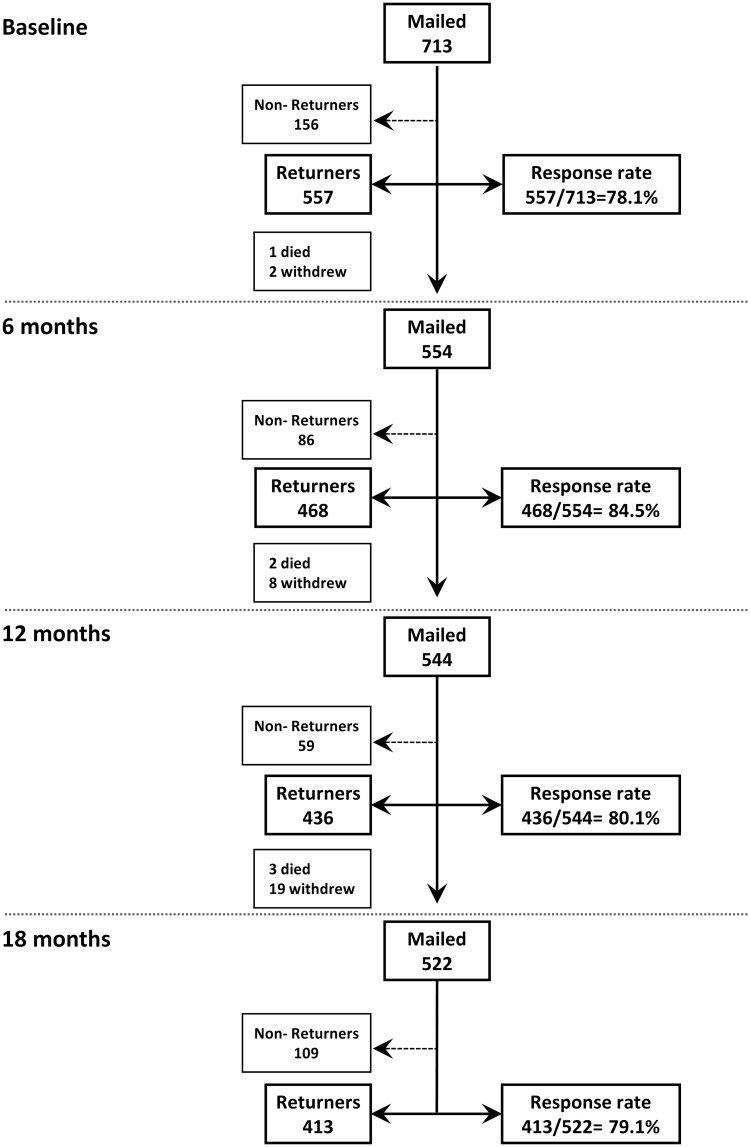
Questionnaire returners and response rates during 18 months of follow-up

Of 713 patients commencing ADA, 557 (78.1%) returned a baseline questionnaire (Fig. 1). A high response rate (>75%) was maintained throughout the follow-up period. No systematic differences were observed between questionnaire returners and non-returners, including gender, age at disease onset and disease activity. More non-returners resided in the most socially deprived quartile [50/156 (32.1%) *vs* 102/557 (18.3%)], although in the final analysis each quartile was well represented (Table 1).

**Table 1:** Descriptive information of complete case baseline data of adalimumab-treated RA patients

Variable	Value
Age at onset, mean (s.d.), years	44.25 (13.26)
Age at registration, mean (s.d.), years	55.92 (12.27)
Female gender, *n* (%)	257/329 (78.1)
Ethnicity, *n* (%)	
White	320/329 (97.3)
Black African	0
Black British	1/329 (0.3)
Indian	3/329 (0.9)
Pakistani	1/329 (0.3)
Bangladeshi	0
Other	4/329 (1.2)
Social deprivation quartile, *n* (%)	
1 (least deprived)	87/329 (26.4)
2	99/329 (30.0)
3	79/329 (24.0)
4 (most deprived)	64/329 (19.4)
Working status, *n* (%)	
Working	125/329 (37.9)
Not working due to illness	84/329 (25.5)
Retired	120/329 (36.6)
Ever smoker, *n* (%), yes/no	190/329 (57.6)
Disease activity	
Number of baseline sDMARDs, *n* (%)	
0	62/329 (18.8)
1	161/329 (48.9)
2	77/329 (23.4)
3	29/329 (8.8)
Disease duration, mean (s.d.), years	11.60 (9.22)
Satisfy ACR criteria, *n* (%), yes/no	275/329 (83.6)
Morning stiffness, *n* (%), yes/no	312/329 (94.8)
Involvement of >3 joints, *n* (%), yes/no	274/329 (83.3)
Involvement of hand joint, *n* (%), yes/no	259/329 (78.7)
Symmetry, *n* (%), yes/no	272/329 (82.7)
Nodules, *n* (%), yes/no	120/329 (36.5)
RF positive, *n* (%), yes/no	217/329 (66.0)
Erosions on X-ray, *n* (%), yes/no	193/329 (58.7)
Swollen joint count (0–28), mean (s.d.)	11 (6)
Tender joint count (0–28), mean (s.d.)	16 (7)
Inflammatory marker, *n* (%), yes/no	177/329 (53.8)
Disease activity score (0–9.3), mean (s.d.)	
Baseline	6.44 (0.94)
6 months (*n* = 295)	3.95 (1.52)
12 months (*n* = 278)	3.77 (1.59)
18 months (*n* = 257)	3.57 (1.53)
DAS patient global score (0–100), mean (s.d.)	73.13 (16.99)
Psychological factors	
IPQ-R domains, mean (s.d.)	
Disease identity (0–14)	6.44 (2.33)
Timeline acute/chronic (6–30)	26.03 (3.28)
Consequences (6–30)	23.18 (3.73)
Personal control (6–30)	18.76 (4.24)
Treatment control (5–25)	17.71 (2.50)
Illness coherence (5–25)	18.63 (3.57)
Timeline cyclical (4–20)	14.67 (2.89)
Emotional (6–30)	19.85 (4.38)
HADS (0–21), mean (s.d.)	
Anxiety	7.57 (4.30)
Depression	6.79 (3.94)
BMQ (5–25), mean (s.d.)	
Necessity	21.54 (2.68)
Concern	14.95 (3.42)
EQ-5D utility score >0.516, *n* (%)	
Baseline	174/329 (52.9)
6 months	231/289 (79.9)
12 months	215/267 (80.5)
18 months	212/256 (82.8)
EQ-5D (baseline) VAS health today (0–100), mean (s.d.)	45.56 (20.72)
EQ-5D (baseline) health in last 12 months, *n* (%)	
Better	33/329 (10.0)
Same	101/329 (30.6)
Worse	195/329 (59.3)
Coping, mean (s.d.)	
Problem focused (4–16)	12 (10, 13)
Emotionally focused (4–16)	10 (8, 12)
Support (family) (2–8)	7 (6, 8)
Support (religion) (1–4)	1 (1, 2)
Functional disability	
HAQ (0–3), mean (s.d.)	
Baseline (*n* = 329)	1.80 (0.62)
6 months (*n* = 273)	1.43 (0.77)
12 months (*n* = 255)	1.43 (0.77)
18 months (*n* = 247)	1.42 (0.77)
SF-36 baseline domains (0–100), mean (s.d.)	
Physical function	26.87 (22.85)
Physical role	25.06 (25.31)
Bodily pain	27.47 (16.89)
General health	32.77 (19.10)
Vitality	25.76 (19.16)
Social	42.63 (24.96)
Emotional	53.17 (34.62)
Mental health	57.51 (20.48)
Physical Component Summary, mean (s.d.)	
Baseline (*n* = 329)	18.72 (9.41)
6 months (*n* = 277)	27.04 (13.02)
12 months (*n* = 255)	27.25 (13.32)
18 months (*n* = 245)	28.13 (13.42)
Mental Component Summary, mean (s.d.)	
Baseline (*n* = 329)	44.12 (11.30)
6 months (*n* = 277)	48.61 (12.53)
12 months (*n* = 255)	50.11 (11.69)
18 months (*n* = 245)	49.60 (11.92)

BMQ: Beliefs about Medicines Questionnaire; EQ-5D: EuroQol five-dimension questionnaire; HADs: Hospital Anxiety and Depression Scale; IPQ-R: Revised Illness Perception Questionnaire; SF-36: 36-item Short Form Health Survey; VAS: visual analogue scale.

Of the 557 patients returning questionnaires, 329 (59.07%) had complete-case data at baseline and were included in the analysis. For each follow-up, those with a complete CQR score contributed to the analysis. The missing CQR scores at follow-up were a combination of incomplete items needed to generate the CQR (6, 12 and 18 month follow-up; *n* = 30, 32 and 33, respectively) or returning a completely unanswered CQR (6, 12 and 18 months follow-up; *n* = 58, 67 and 88, respectively). More than 50% of CQR scores were available in individuals switching or stopping medication, with no pattern observed in those with or without a CQR score during follow-up (supplementary Table S1, available at *Rheumatology* Online). The imputed data consisted of 556 individuals (one observation omitted because it was a severe outlier) and 40 imputations over baseline and 3 follow-ups.

In the complete-case dataset at baseline there were 257/329 (78%) women with a mean age at symptom onset of 44 years (s.d. 13) (Table 1). The majority were of white British ethnicity and across all levels of social deprivation (between 20% and 30% in each quartile). Two hundred and fourteen (62.1%) were not working due to either illness or retirement and 190 (57.1%) had ever smoked.

The mean CQR score remained <75 over the follow-up. After dividing CQR scores into quartiles, 56 (23.2%), 54 (23.7%) and 48 (23.3%) patients for 6, 12 and 18 months, respectively, had adherence scores in the lowest quartile (CQR score 40–64) (Table 2). Variability was noted in individuals’ adherence score over time, such that 41% of those returning all follow-ups (*n* = 59/143) reported a CQR score of 40–64 at least once over the course of the follow-up.

**Table 2:** Adherence as indicated by the CQR score over the follow-up

Follow-up period, months	CQR total score, mean (s.d.)	CQR quartiles, *n* (%)			
		Least adherent		Most adherent	
		40–64	65–74	75–83	84+
6 (*n* = 241)	74.99 (10.40)	56 (23.24)	57 (23.65)	70 (29.05)	58 (24.07)
12 (*n* = 228)	74.96 (10.75)	54 (23.68)	57 (25.00)	57 (25.00)	60 (26.32)
18 (*n* = 206)	74.59 (10.54)	48 (23.30)	60 (29.13)	52 (25.24)	46 (22.33)

CQR: Compliance Questionnaire for Rheumatology.

### Influence of demographic factors

Older age was the only demographic factor significantly associated with an increase in the CQR score [β coefficient 0.14 (s.e. 0.04), *P* = 0.001] (Table 3).

**Table 3:** Univariate random intercept models reflecting the predictors’ influence on CQR score in ADA-treated patients

Predictor		β_0_ intercept constant, coefficient (s.e.)	β_1_, coefficient (s.e.)	Random intercept variance, estimate (s.e.)	Overall error (residual variance), estimate (s.e.)
Demographic					
Age at questionnaire, years		67.19 (2.64)	0.14 (0.04)**	63.44 (6.17)	40.79 (2.12)
Gender, female		67.19 (2.64)	0.76 (1.19)	63.44 (6.17)	40.79 (2.12)
Social deprivation					
Quartile 2 (least deprived)		67.19 (2.64)	1.40 (1.03)	63.44 (6.17)	40.79 (2.12)
Quartile 3		67.19 (2.64)	−0.11 (1.39)	63.44 (6.17)	40.79 (2.12)
Quartile 4 (most deprived)		67.19 (2.64)	1.36 (1.48)	63.44 (6.17)	40.79 (2.12)
Ever smoked		66.99 (2.66)	0.43 (1.01)	63.29 (6.16)	40.86 (2.22)
Disease activity					
Number of baseline sDMARDs		66.60 (2.85)	0.30 (0.58)	63.19 (6.16)	40.88 (2.22)
Disease duration		67.19 (2.64)	−0.14 (0.06)*	63.44 (6.17)	40.79 (2.12)
Satisfy ACR criteria		67.72 (2.80)	−0.78 (1.34)	63.17 (6.15)	40.88 (2.22)
Morning stiffness		64.89 (3.36)	2.43 (2.29)	63.03 (6.14)	40.87 (2.22)
Involvement in >3 joints		68.25 (2.83)	−1.37 (1.32)	63.08 (6.14)	40.86 (2.22)
Involvement in hand joint		68.82 (2.77)	−2.17 (1.20)	62.49 (6.10)	40.88 (2.22)
RF positive		66.96 (2.73)	0.30 (1.04)	63.28 (6.16)	40.87 (2.22)
Erosions on X-ray		67.14 (2.67)	0.04 (1.03)	63.28 (6.16)	40.87 (2.22)
DAS28		68.04 (2.76)	−0.13 (0.14)	63.08 (6.32)	41.30 (2.43)
Presence of inflammation		67.50 (2.68)	−0.68 (0.67)	62.45 (6.32)	41.68 (2.47)
Functional disability					
HAQ		66.83 (2.65)	0.09 (0.49)	63.19 (6.17)	39.99 (2.24)
SF-36 domains					
Physical function		67.34 (2.77)	−0.002 (0.01)	63.87 (6.23)	40.47 (2.23)
Physical role		67.10 (2.68)	0.002 (0.009)	63.89 (6.23)	40.48 (2.23)
Bodily pain		66.86 (2.69)	0.007 (0.01)	63.83 (6.23)	40.48 (2.23)
General health		66.89 (2.68)	0.003 (0.01)	63.81 (6.21)	40.27 (2.20)
Vitality		66.87 (2.67)	0.01 (0.01)	63.61 (6.19)	40.58 (2.21)
Social		67.01 (2.70)	0.003 (0.01)	63.68 (6.23)	40.64 (2.25)
Emotional		66.83 (2.72)	0.004 (0.009)	63.47 (6.21)	40.68 (2.25)
Mental health + time interactions		66.84 (2.90)	0.005 (0.02)	63.05 (6.13)	40.00 (2.18)
Fup1*mental health	Interaction χ^2^ = 11.12, *P* = 0.01	–	−0.03 (0.03)	–	–
Fup2*mental health		–	0.05 (0.03)	–	–
Fup3*mental health		–	0.06 (0.03)*	–	–
Physical Component Summary		66.82 (2.77)	−0.0003 (0.03)	63.90 (6.26)	40.42 (2.25)
Mental Component Summary		65.60 (2.89)	0.03 (0.03)	63.84 (6.25)	40.38 (2.25)
Psychological					
IPQ-R					
Disease identity		69.02 (2.93)	−0.31 (0.21)	62.83 (6.12)	40.87 (2.22)
Timeline acute/chronic		52.24 (4.81)	0.56 (0.15)**	60.36 (5.92)	40.83 (2.22)
Consequences		61.49 (4.18)	0.23 (0.13)	62.60 (6.10)	40.87 (2.22)
Personal control		66.24 (3.49)	0.05 (0.12)	63.26 (6.16)	40.87 (2.22)
Treatment control		55.18 (4.45)	0.67 (0.20)**	60.73 (5.96)	40.88 (2.22)
Illness coherence		56.71 (3.91)	0.49 (0.14)**	60.42 (5.93)	40.86 (2.22)
Timeline cyclic		65.94 (3.56)	0.09 (0.17)	63.24 (6.16)	40.87 (2.22)
Emotional representation		63.11 (3.61)	0.19 (0.11)	62.54 (6.11)	40.90 (2.22)
HADS					
Anxiety + time interaction		67.82 (2.83)	−0.09 (0.13)	63.27 (6.15)	40.38 (2.19)
Fup1*anxiety	Interaction χ^2^ = 8.11, *P* = 0.04	–	0.32 (0.13)*	–	–
Fup2*anxiety		–	−0.04 (0.14)	–	–
Fup3*anxiety		–	0.05 (0.14)	–	–
Depression		68.07 (2.78)	−0.13 (0.13)	63.04 (6.14)	40.87 (2.22)
BMQ					
Necessity + time interaction		27.20 (4.77)	1.88 (0.19)**	48.16 (4.89)	39.77 (2.15)
Fup1*necessity	Interaction *X*^2^ = 16.12, *P* = 0.001	–	−0.43 (0.21)*	–	–
Fup2*necessity		–	−0.54 (0.22)*	–	–
Fup3*necessity		–	−0.84 (0.22)**	–	–
Concern + time interaction		77.20 (3.64)	−0.64 (0.16)**	60.34 (5.91)	40.26 (2.19)
Fup1*concern	Interaction *X*^2^ = 10.66, *P* = 0.014	–	0.52 (0.17)**	–	–
Fup2*concern		–	0.17 (0.17)	–	–
Fup3*concern		–	−0.008 (0.17)	–	–
EQ-5D					
Health today		67.68 (2.71)	−0.01 (0.01)	62.95 (6.15)	41.08 (2.24)
Utility group > 0.516		67.01 (2.67)	0.32 (0.59)	63.21 (6.18)	41.01 (2.24)
Coping					
Problem focused		64.91 (3.40)	0.19 (0.18)	62.97 (6.14)	40.89 (2.22)
Emotionally focused		65.86 (3.08)	0.16 (0.19)	63.15 (6.15)	40.87 (2.22)
Family/professional support		59.65 (3.40)	1.25 (0.37)**	60.64 (5.95)	40.87 (2.22)

*P < 0.05; **P < 0.005.

ADA: adalimumab; BMQ: Beliefs about Medicines Questionnaire; CQR: Compliance Questionnaire for Rheumatology; DAS28: 28-joint DAS; EQ-5D: EuroQol five-dimensions questionnaire; Fup1: follow up at 6 months; Fup2: follow up at 12 months; Fup3: follow up at 18 months; HADs: Hospital Anxiety and Depression Scale; IPQ-R: Revised Illness Perception Questionnaire; SF-36: 36-item Short Form Health Survey.

### Influence of disease activity and physical disability

Concomitant sDMARD treatment with ADA was used in 287 (81.2%) patients. Individuals had a mean disease duration of 11.6 years (s.d. 9.2). They had a high mean baseline DAS28 score of 6.4 (s.d. 0.9) and 177 (53.8%) had an increased acute phase response. At 6 months the DAS28 score was 3.95 (s.d. 1.5), with this response maintained over the follow-up. In parallel, the HAQ and SF-36 Physical Component Summary (PCS) scores improved over time with response to treatment (Table 1). The DAS28, high acute phase reactants and HAQ score did not predict the CQR score over time, although longer disease duration was associated with a lower adherence score [β_1_ coefficient −0.15 (s.e. 0.06), *P* = 0.009] (Table 3).

### Influence of illness cognitions and mood

From the illness perception measures, there were high mean scores in patients’ awareness of the long-lasting nature of RA [timeline 26.0 (s.d. 3.28)], with this domain also predicting increased levels of adherence [β coefficient 0.56 (s.e. 0.15), *P* = 0.0001] (Tables 1 and 3). Patients had a high coherent understanding of their illness [mean 18.6 (s.d. 3.57)] and treatment control [mean 18.8 (s.d. 4.24)], with both baseline effects significantly increasing the CQR score [β_1_ coefficient 0.49 (s.e. 0.14), *P* = 0.0001 and 0.67 (s.e. 0.2), *P* = 0.001, respectively]. Individuals sought more professional/family support compared with other coping strategies {median 7 [interquartile range (IQR) 6–8]} (Table 1). This support was associated with an increase in expected adherence [β_1_ coefficient 1.25 (s.e. 0.37), *P* = 0.001] (Table 3). Medication necessity was high [mean 21.54 (s.d. 2.68)] and had an increasing effect on the CQR score [β_1_ coefficient 1.88 (s.e. 0.19), *P* = 0.0001)]. Increased medication concern was associated with a reduced CQR score [β_1_ coefficient −0.64 (s.e. 0.16), *P* = 0.0005)], with a significant increase in this effect between baseline and 6 months [0.52 (s.e. 0.17), *P* < 0.005] that remained during further follow-up (Table 3).

In the imputed dataset, the importance of medication beliefs was also observed in the univariate analysis. Perceived health in the previous 12 months, utility score (EQ-5D) and SF-36 PCS and Mental Component Summary (MCS) scores were also significant univariately, although with low coefficients (supplementary Table S2, available at *Rheumatology* Online). Anxiety and depression scores from the HADS were not predictive of the adherence score (Table 3).

### Independent predictors of adherence

In a multivariate analysis of the CC and imputed (IM) datasets it was found that an increased belief in medication necessity was a significant independent predictor of adherence in both the CC [1.68 (s.e. 0.19), *P* = 0.0001] and IM datasets [1.65 (s.e. 0.18), *P* = 0.0001], with this baseline effect on the CQR score diminishing over time, as indicated by the significant negative time interaction (Table 4). High medication concerns were also predictive of a lower CQR score [CC = −0.50 (s.e. 0.15), *P* = 0.001; IM = −0.49 (s.e. 0.14), *P* = 0.05], with the effect remaining important throughout follow-up. Increased professional or family support was associated with an increased CQR score [CC = 0.81 (s.e. 0.32), *P* = 0.01; IM = 0.87 (s.e. 0.28), *P* = 0.002]. A stronger perceived view of their illness being chronic [0.32 (s.e. 0.14), *P* = 0.025] and an increased feeling of treatment control [0.41 (s.e. 0.19), *P* = 0.032] at baseline also predicted an increased CQR score in the CC dataset. Comparing the random intercept variance of the final model and unconditional model [63.23 (s.e. 6.16) and 43.02 (s.e. 4.46), respectively] showed a potential 20% of individual variance in CQR score being accounted for by medication and illness beliefs and the patient adopting coping strategies at baseline. Weakened influence of an individual’s baseline perception of their chronicity of illness (timeline domain) and their perceived treatment control on adherence over time were seen in the imputed dataset.

**Table 4:** Final random intercept models of independent predictors of CQR over time in adalimumab-treated patients

Factors associated with CQR^a^	Complete case model	Imputed model
	β coefficient (s.e.)	β coefficient (s.e.)
Necessity	1.68 (0.19)**	1.65 (0.18)**
Fup1*necessity	−0.44 (0.21)*	−0.46 (0.20)*
Fup2*necessity	−0.54 (0.21)*	−0.58 (0.20)**
Fup3*necessity	−0.86 (0.22)**	−0.64 (0.21)**
Concern	−0.50 (0.15)**	−0.49 (0.14)**
Fup1*concern	0.50 (0.17)**	0.31 (0.16)*
Fup2*concern	0.14 (0.17)	0.33 (0.16)*
Fup3*concern	−0.05 (0.17)	0.04 (0.17)
Support	0.81 (0.32)*	0.87 (0.28)**
Timeline acute/chronic	0.32 (0.14)*	0.24 (0.13)
Treatment control	0.41 (0.19)*	0.27 (0.17)
Fup1	1.53 (5.17)	5.43 (4.84)
Fup2	9.55 (5.25)	8.84 (4.87)
Fup3	18.92 (5.48)**	14.67 (5.32)*
β_0_ intercept constant	18.44 (7.45)	23.00 (6.68)
${\text{σ}}_{\text{u}0}^{\text{2}}$ intercept variance (s.e.)	43.02 (4.46)	48.86 (4.82)
${\text{σ}}_{\text{E}}^{\text{2}}$ overall error (residual) (s.e.)	39.18 (2.11)	55.92 (3.13)

^a^Adjusted for age, gender, disease duration and social deprivation. *P < 0.05; **P < 0.005. CQR: Compliance Questionnaire for Rheumatology; Fup1: follow up at 6 months; Fup2: follow up at 12 months; Fup3: follow up at 18 months.

To better understand the influence of medication beliefs, we utilized the final CC model to determine predicted CQR scores over time using extreme values of necessity and concern responses. Individuals showing acceptance towards their medication (high necessity and low concern) had a predicted CQR score of 78.7 (s.e. 2.0) at 6 months compared with 54.0 (s.e. 4.1) for sceptical individuals (low necessity and high concern) (Table 5).

**Table 5:** Predictive margins of CQR score during follow-up for levels of an individuals’ treatment belief

Individual	Baseline BMQ domain score		Predicted CQR score (95% CI)		
	Necessity	Concern	6 months	12 months	18 months
Accepting	25	5	78.74 (74.81, 82.67)	82.50 (78.53, 86.48)	82.89 (78.97, 86.82)
Ambivalent	25	25	78.85 (75.00, 82.69)	75.40 (71.47, 79.33)	71.93 (67.89, 75.96)
Indifferent	5	5	53.89 (46.07, 61.72)	59.62 (51.67, 67.56)	66.42 (58.17, 74.67)
Sceptical	5	25	54.00 (45.87, 62.11)	52.52 (44.23, 60.81)	55.46 (47.32, 63.60)

BMQ: Beliefs about Medicines Questionnaire; CQR: Compliance Questionnaire for Rheumatology.

## Discussion

This study is one of the first longitudinal studies assessing adherence to biologic therapy in RA patients using a self-reported measure. More than 50% of individuals had a CQR score <75, indicating compromised adherence. The levels of adherence found were comparable to previously reported rates for oral sDMARDs using the same CQR adherence measure [3, 4, 6, 20] and also support observations of low adherence measured by ADA possession ratio or persistence [7–9]. To our knowledge, this is the first study to investigate the relative contribution of demographic factors and RA disease-specific and psychological behavioural influences on adherence in a single prospective cohort.

Increased perception of treatment control as an independent predictor of increased adherence was indicative of patients starting an injectable drug such as ADA and retaining some sense of treatment control and high expectations of the new medication [46]. The association of coping through seeking professional/family support is verified by findings from a meta-analysis of 122 studies published between 1948 and 2001. Patients receiving social support were 3.6 times more likely to adhere to medication than those not receiving support [47]. The support may reflect the intense pretreatment counselling or indicate patients moving into a more dependent phase of their illness. However, little is known about the most effective type of coping and the association with adherence.

A key observation in our RA cohort was the major influence of patient beliefs on treatment adherence, seen also in other chronic conditions including asthma, hypertension and chronic pain [23]. The necessity-concern framework [25] suggests that a patient’s adherence decisions are a result of the balance between their perceived need for the medication (necessity) and their concerns regarding its use. We were able to stratify individuals into specific treatment attitudes based on necessity and concern and estimate the potential influence that combinations of beliefs have on adherence. The importance of medication necessity for new users of ADA regardless of concern level was fundamental in predicting increased adherence and requires further work in establishing the stability of both beliefs over the treatment and disease pathway.

Several studies of oral sDMARD adherence, as well as studies in other conditions, have also shown similar importance of medication beliefs [4, 5, 23]. Non-adherence may therefore owe more to individual patient beliefs than to the actual disease or route of drug administration. A patient’s level of medication belief may of course be influenced by the perceived intensity of the drug and/or its mode of administration. However, our data suggest that the influence on adherence remains qualitatively similar across therapy types.

Our findings reflect those of other studies showing that older age is associated with higher adherence [14]. Others have found that increased age predicted early termination of biologic therapy [10], although persistence studies may measure stopping due to efficacy or adverse events rather than adherence.

Measures of disease activity and functional disability were not associated with adherence in our study, despite high levels of disease activity and high HAQ scores at baseline. Others have also noted that neither disease duration nor disease activity (using ESR and CRP level) were associated with adherence in RA [5]. In contrast, Owen *et al.* [48] found increased ESR and morning stiffness were associated with higher adherence in a univariate analysis (as measured by interview). Our patients had established disease and had a high perceived understanding of the cyclical nature of the disease, a high level of illness coherence and adopted a large number of coping strategies. This long-standing experience of RA may have made them more aware of the implications of further flares and thus to keep taking medication even when feeling better. Also, our assessment of disease activity and disability was sampled over a short period of their overall disease experience. As such, it may have little effect on whether a patient chooses to take his/her medication at a specific time.

Our findings have important implications not only on ultimately reducing the economic impact of non-adherence to biologic therapies, but also in improving routine clinical outcomes. First, it highlights the need for clinicians to be vigilant for potential non-adherence in patients taking biologic therapies. It also highlights the important role of perceived health care support an individual receives, thus the supportive and empathic aspects of clinical practice should be enhanced. Our study has identified potential modifiable patient beliefs. Clinicians therefore need to address the patient’s perception of medication need and concerns early in the treatment course. Further, the diminishing effect of the necessity belief over follow-up suggests that the need for medication should be reiterated throughout the treatment course. There is, however, a clear dilemma at an individual level of how patients judge personal need relative to the concern about their medication which influences the motivation of taking the medication. More than 40% of patients in this study had a strong belief in the need for treatment but simultaneously expressed strong concerns about medication use. Non-adherence is often the response to the latter. Identifying and targeting this at-risk group may be of particular value in improving overall clinical outcomes. With the increasing use of biologic therapies, approaches to improve adherence will likely reduce the economic burden by reducing wastage of drugs and avoid further drug escalation. Early evidence has shown promising potential for an SR-CSM-related behaviour intervention to target key cognitions [49]. Such inventions may be feasible in a routine clinical consultation to improve adherence and thus overall outcomes.

A study such as this has potential limitations. Missing data are inevitable in large observational studies using postal questionnaires. The majority of measures used were composite scores, and unless the initial methodology included a way to handle missing responses, the total score was marked as missing. The analysis of missing data only indicated that younger individuals had more complete data. Patterns of missing data showed an absence of monotone pattern and was indicative of an arbitrary one, which was effectively approached using Multiple Imputation for Chained Equations where appropriate. In addition, the mixed model methodology also allowed all available follow-up data to be utilized without missing scores having any effect on other available scores for the same individual.

Accurately defining and measuring adherence is difficult. Some of the CQR items incorporating attitudinal constructs related to medication taking may potentially confound the relationship between the medication belief scale and adherence. At the time of study, the CQR was the only validated RA adherence questionnaire.

Our study focused on patients starting ADA, because of the time period in which patients were recruited from the BSRBR-RA. There is some evidence that medication possession ratios and persistence rates differ across biologic therapies [8, 10, 11]. However, those studies did not use the measures employed in this study, so observed variability may be due to adverse event profiles or efficacy differences. However, our results do accord with observations in other chronic disease, suggesting they may be generalized to other ambulatory drugs in RA. It is also possible that our analyses were limited by illness and medication beliefs not being captured beyond the baseline visit. Few longitudinal studies have investigated the stability of beliefs over time, although general beliefs in non-prescribed analgesics have been shown to be stable over time [50]. Thurah *et al.* [4] also noted in 65 new users of MTX that concern levels were stable over a 9-month period. Further larger longitudinal work addressing changes in treatment beliefs would be advantageous to inform on patients behavioural influences over time. In addition, co-morbidities were not considered, which may further impact on adherence by the increasing number of medication regimes and the choice of one regime over another for multiple conditions, thus influencing illness and medication concerns. Finally, the nature of the study also prevented recording the influence of the patient–consultant relationship and contact time, the provision and extent of medication and disease information and other patient factors such as self-efficacy, which may be additional influences on medication adherence.

In conclusion, a quarter of patients showed only low to moderate adherence (CQR score <65) to ADA, a self-administered injectable biologic therapy. Medication beliefs (high concerns and low necessity beliefs) were associated with lower adherence. Increased professional/family support, stronger perceived illness chronicity and an increased feeling of treatment control also predicted adherence over time. These findings highlight the need to prioritize the monitoring of biologic treatment adherence in a chronic symptomatic disease group such as RA.

## Supplementary Material

Supplementary Data

## Notes

*Funding*: This study was funded by Arthritis Research UK Centre of Epidemiology Core Grant 17 552.

*Disclosure statement*: I.N.B. holds grants from UCB, Sanofi/Genzyme, Roche and GlaxoSmithKline and honoraria/speakers bureaus from UCB, GlaxoSmithKline, Roche, Pfizer and Medimmune outside of the submitted work. L.C. has received honoraria from Janssen and AbbVie for educational events and has received an unrestricted research award as a co-applicant from Pfizer. None of these awards are associated with the submitted manuscript. K.L.H. has received honoraria from Pfizer and AbbVie. All other authors have declared no conflicts of interest.
