# Supplementary material for: The influence of behavioural and psychological factors on medication adherence over time in rheumatoid arthritis patients: a study in the biologics era
Source: Rheumatology (Oxford). 2015 May 12;54(10):1780–91. doi: 10.1093/rheumatology/kev105 (PMC4571488; doi:10.1093/rheumatology/kev105)
Supplement: Supplementary Data [file supp_54_10_1780__index.html]

The influence of behavioural and psychological factors on medication adherence over time in rheumatoid arthritis patients: a study in the biologics era — The influence of behavioural and psychological factors on medication adherence over time in rheumatoid arthritis patients: a study in the biologics era — Supplementary Data 

# The influence of behavioural and psychological factors on medication adherence over time in rheumatoid arthritis patients: a study in the biologics era

## Supplementary Data

files

**Files in this Data Supplement:**

- Supplementary Data - pdf file
