# Supplementary material for: The influence of behavioural and psychological factors on medication adherence over time in rheumatoid arthritis patients: a study in the biologics era
Source: Rheumatology (Oxford). 2015 May 12;54(10):1780–91. doi: 10.1093/rheumatology/kev105 (PMC4571488; doi:10.1093/rheumatology/kev105)
Supplement: Supplementary Data [file supp_kev105_rhe-14-1223.pdf]

Supplementary Table S1: Proportion of individuals stopping or switching adalimumab by missing follow up CQR score.

**Stopped ADA < 6 months N=46**	**Stopped a biologic altogether N=24**				**Switched N=22**		
	Ineffective n=10	Adverse event n=10	Other n=4	Missing reason n=0	Ineffective n=13	Adverse event n=8	Missing reason n=1
Missing CQR at follow up							
1	2/10	2/10	1/4	0	4/13	1/8	0/1
2	7/10	4/10	1/4	0	8/13	2/8	0/1
3	4/10	5/10	3/4	0	5/13	2/8	0/1
**Stopped ADA 6-12 months, N=31**	**Stopped a biologic altogether N=24**				**Switched**	**N=7**	
	Ineffective n=12	Adverse event n=9	Other n=3	Missing reason n=0	Ineffective n=4	Adverse event n=2	Missing reason n=1
Missing CQR at follow up							
1	5/12	5/9	1/3	0	0/4	0/2	0/1
2	4/12	4/9	1/3	0	1/4	0/2	0/1
3	6/12	3/9	2/3	0	1/4	0/2	0/1
**Stopped ADA 12-18 months, N=20**	**Stopped a biologic altogether N=12**				**Switched N=8**		
	Ineffective n=3	Adverse event n=3	Other n=5	Missing reason n=1	Ineffective n=5	Adverse event n=2	Missing reason n=1
Missing CQR at follow up							
1	1/3	1/3	1/5	1/1	1/5	1/2	0/1
2	2/3	1/3	3/5	1/1	2/5	1/2	0/1
3	2/3	2/3	2/5	1/1	2/5	0/2	0/1

ADA: adalimumab; CQR: Compliance Questionnaire- Rheumatology.

Supplementary Table S2: Univariate random intercept models of imputed data reflecting the predictors' influence on CQR score in ADA treated patients

Predictor[a]	β_0 intercept constant Coefficient (SE)	β_1 Coefficient (SE)	Random intercept variance Estimate (SE)	Overall error (residual variance) Estimate (SE)
**Demographic**				
Age at questionnaire (yr)	66.98 (2.42)	0.13 (0.04)**	67.41 (5.81)	57.72 (3.28)
Gender (female)	66.98 (2.42)	-0.41 (1.01)	67.41 (5.81)	57.72 (3.28)
Social deprivation (high) quartile 2	66.98 (2.42)	1.95 (1.10)	67.41 (5.81)	57.72 (3.28)
quartile 3	66.98 (2.42)	1.64 (1.13)	67.41 (5.81)	57.72 (3.28)
(low) quartile 4	66.98 (2.42)	2.12 (1.29)	67.41 (5.81)	57.72 (3.28)
Ever smoked	66.64 (2.45)	0.59 (0.87)	67.45 (5.82)	57.73 (3.28)
**Disease activity**				
Number of baseline DMARDs	67.79 (2.65)	-0.39 (0.49)	67.39 (5.79)	57.73 (3.28)
Disease duration	66.98 (2.42)	-0.05 (0.05)	67.41 (5.81)	57.72 (3.28)
Satisfy ACR criteria	67.53 (2.55)	-0.76 (1.14)	67.48 (5.82)	57.72 (3.28)
Morning stiffness	64.35 (2.93)	2.79 (1.91)	67.07 (5.77)	57.72 (3.28)
Involvement in >3 joints	67.99 (2.55)	-1.28 (1.11)	67.34 (5.82)	57.72 (3.28)
Involvement in hand joint	67.81 (2.52)	-1.13 (1.02)	67.35 (5.80)	57.72 (3.28)
RF positive	66.52 (2.48)	0.74 (0.88)	67.39 (5.83)	57.72 (3.28)
Erosions on x-ray	66.82 (2.46)	0.31 (0.91)	67.48 (5.83)	57.73 (3.28)
Disease activity score (DAS28)	68.17 (2.53)	-0.28 (0.13)*	67.49 (5.80)	57.49 (3.27)
Presence of inflammation	66.46 (2.48)	1.06 (0.85)	67.23 (5.80)	57.73 (3.28)
**Functional disability**				
HAQ[b]	67.23 (2.43)	-0.46 (0.50)	67.79 (5.80)	57.59 (3.27)
SF-36 domains[c]				
Physical function	64.88 (2.52)	0.03 (0.01)*	67.60 (5.79)	57.24 (3.28)
Physical role	65.84 (2.47)	0.02 (0.009)*	67.55 (5.81)	57.40 (3.26)
Bodily pain	65.40 (2.48)	0.03 (0.01)**	67.41 (5.80)	57.19 (3.26)
General health	65.88 (2.48)	0.03 (0.02)*	68.15 (5.84)	57.26 (3.28)
Vitality	65.98 (2.48)	0.03 (0.01)*	68.01 (5.84)	57.28 (3.34)
Social	65.54 (2.51)	0.02 (0.009)*	67.74 (5.81)	57.31 (3.27)
Emotional	65.63 (2.56)	0.02 (0.009)*	67.47 (5.81)	57.46 (3.28)
Mental health	64.20 (2.57)	0.05 (0.01)**	67.48 (5.82)	57.10 (3.30)
physical component summary	64.75 (2.54)	0.07 (0.02)*	67.75 (5.79)	57.21 (3.25)
mental component summary	64.16 (2.75)	0.06 (0.03)*	67.74 (5.83)	57.28 (3.32)
**Psychological**				
IPQ-R[d]				
disease identity	67.43 (2.70)	-0.07 (0.20)	67.47 (5.82)	57.72 (3.28)
timeline acute/chronic	53.85 (4.22)	0.50 (0.13)**	64.78 (5.65)	57.74 (3.28)
Consequences	62.72 (3.73)	0.17 (0.11)	67.14 (5.79)	57.72 (3.28)
personal control	66.94 (3.24)	0.002 (0.10)	67.52 (5.82)	57.72 (3.28)
treatment control	58.40 (3.95)	0.48 (0.17)*	66.17 (5.75)	57.71 (3.28)
illness coherence	60.50 (3.43)	0.31 (0.12)*	66.17 (5.80)	57.72 (3.28)
timeline cyclic	65.40 (3.26)	0.11 (0.14)	67.42 (5.81)	57.72 (3.28)
emotional representation	65.19 (3.30)	0.08 (010)	67.42 (5.81)	57.72 (3.28)
HADs[e]				

Anxiety	66.63 (2.56)	0.04 (0.10)	67.51 (5.81)	57.72 (3.28)
Depression	66.89 (2.53)	0.01 (0.11)	67.52 (5.81)	57.72 (3.28)
BMQ[f]				
necessity	37.16 (4.05)	1.38 (0.15)**	53.62 (5.12)	57.71 (3.28)
Concern	73.05 (3.17)	-0.37 (0.12)**	65.76 (5.77)	57.74 (3.28)
EQ-5D[g]				
health today	65.41 (2.51)	0.03 (0.01)*	67.41 (5.82)	57.41 (3.25)
utility group >0.516	65.48 (2.48)	1.88 (0.56)**	68.21 (5.83)	56.97 (3.27)
Coping[h]				
problem focused	63.97 (3.04)	0.26 (0.15)	66.95 (5.82)	57.73 (3.28)
emotionally focused	65.73 (2.80)	0.15 (0.16)	67.36 (5.81)	57.73 (3.28)
family/professional support	59.86 (3.02)	1.25 (0.31)**	64.57 (5.66)	57.74 (3.28)

*p<0.05; **p<0.005. [a]Adjusted for age at follow up , gender, disease duration and social deprivation or in combinations where appropriate. [b]HAQ: higher score indicative of greater disability; [c]SF-36: higher score indicative of better health status; [d]IPQ-R: higher score across domains are indicative of greater sense of symptomology; acute long-lasting nature of disease; cyclical nature of disease; ultimate consequences of disease; own personal control or treatment control over disease; understanding of disease; high emotional state; [e]HADs: higher score indicative of higher emotional state; [f]BMQ: higher score indicative of stronger feeling of medication need and more concern towards medication use; [g]EQ-5D: higher utility score e indicates better patient reported health state; [h]Coping: higher level more coping strategies adopted. β_0 intercept constant; $\beta_{1;}$ Random intercept variance; Overall error (residual variance). SF-36: Short Form Health Survey; EQ-5D: EuroQol-5D; HADs: Hospital Anxiety and Depression scale; BMQ: Beliefs about Medicines Questionnaire; IPQ-R: Revised Illness Perception Questionnaire.
